# Anti-NMDA-receptor encephalitis presenting with catatonia and neuroleptic malignant syndrome in patients with intellectual disability and autism

**DOI:** 10.1192/pb.bp.112.041954

**Published:** 2015-02

**Authors:** Reza Kiani, Mark Lawden, Penelope Eames, Peter Critchley, Sabyasachi Bhaumik, Sunita Odedra, Rohit Gumber

**Affiliations:** 1 Adult Learning Disability Service, Leicestershire Partnership NHS Trust; 2 Department of Medical Education, School of Medicine, University of Leicester; 3 Department of Neurology, University Hospitals of Leicester; 4 University of Leicester

## Abstract

We report anti-*N*-methyl-d-aspartate (NMDA) receptor encephalitis in two patients with autism and intellectual disability presenting with neuropsychiatric symptoms of catatonia and neuroleptic malignant syndrome. Case reports such as these help raise awareness of this clinical issue. By paving the way for earlier diagnoses they ultimately maximise the potential for curative treatments and prevention of long-term complications.

Anti-*N*-methyl-d-aspartate (NMDA) receptor encephalitis is a recently recognised syndrome, which can be mistaken for psychiatric disorders. It is potentially reversible with appropriate treatment. It is usually idiopathic but can be associated with tumours, particularly ovarian teratomas in female patients. Surgical excision of the tumour (if present) and immunotherapy are currently considered optimum treatment for anti-NMDA-receptor encephalitis.

An increasing recognition has been given to the aetiological role of the immune system in the pathogenesis of various psychiatric disorders and early diagnostic tests have been recommended to rule out autoimmunity.^1–3^ Anti-NMDA-receptor encephalitis is one of the recently described autoimmune encephalitides^4^ with a prominently psychiatric presentation.^5^ It is important to diagnose and treat anti-NMDA-receptor encephalitis. Untreated, it can lead to cognitive deficit and death.^6^ Definitive diagnosis relies on the detection of anti-NMDA-receptor antibodies, using a highly specific and sensitive assay, yielding no false positives as yet.^7^ The reduction in NMDA-receptor antibodies with clinical improvement supports the pathogenic role of antibodies in anti-NMDA-receptor encephalitis.^8^

A publication in the *British Journal of Psychiatry* describes four cases of anti-NMDA-receptor encephalitis initially presenting with signs and symptoms suggestive of a psychiatric disorder.^9^ We report two cases of anti-NMDA-receptor encephalitis presenting with catatonia and neuroleptic malignant syndrome (NMS) in individuals with autism and intellectual disability.

## Case studies

### Case study 1

A 32-year-old single woman with a diagnosis of mild intellectual disability, autism and Larsen’s syndrome presented with social withdrawal and a persistently low mood. Subsequently, her sleep and oral intake deteriorated. A preliminary diagnosis of depression was made and she was put on an antidepressant. Unfortunately, her skills deteriorated further and her parents had to attend to her basic needs. She then showed objective evidence of hallucinations when she would shout incoherently or burst into laughter for no apparent reason. She was therefore started on a low-dose antipsychotic medication but then developed incontinence, muteness and rigidity. She maintained a sedentary position and needed a wheelchair to be moved around. There were classic catatonic signs and symptoms such as negativism, echolalia and posturing. At this stage NMS was suspected as the patient had low urine output, low-grade fever, clammy skin and fluctuation of blood pressure. Her psychotropic medication was stopped and she was urgently admitted for further assessment. Investigations revealed a normal creatine kinase (CK) level and no evidence of infection or metabolic imbalance. With many presenting symptoms being of a psychiatric nature, an initial diagnosis of functional catatonia was thought most appropriate. Consequently, treatment with psychotropic medication was reinitiated targeting the psychotic and mood symptoms. Despite this, her condition continued to deteriorate. She then had at least two episodes of non-convulsive seizures (with inter-ictal electrocardiogram (EEG) not showing any evidence of epileptiform activities) and several episodes of what were initially thought to be breath-holding attacks. A referral to the respiratory department ruled out bronchomalacia reported in Larsen’s syndrome. As her oxygen saturation and chest X-ray were normal, these episodes were first thought to be behavioural in nature. She later developed a rabbit-like movement of the lips, which lasted for 2 weeks. Magnetic resonance imagining (MRI) scans of the head and spine were normal.

Anti-NMDA-receptor encephalitis was suspected in view of her complex presentation and normal investigations. A serum sample later on in the course of illness was strongly positive for anti-NMDA-receptor antibodies. Following diagnosis, a thorough investigation was undertaken to rule out the presence of an underlying tumour, particularly an ovarian teratoma, which has been reported in a considerable number of cases of anti-NMDA-receptor encephalitis in females.^10,11^ Abdominal and pelvic ultrasound, brain MRI and chest, abdominal and pelvic computed tomography (CT) scans, blood tests for tumour markers and other autoimmune disorders revealed no abnormalities. By the time she received intravenous methylprednisolone, she had started to show improvement and this gradually continued with the second course.

During recovery she suffered mild amnesia. She would often be searching for something that she was actually holding in her hand, for instance a mobile telephone or spectacles. Owing to a prolonged period of immobility, she developed a flexion contraction in her upper and lower limbs, which improved partially with physiotherapy. Six months later, she had completely recovered with no evidence of psychosis or cognitive deficit and all her skills, apart from ability to walk, returned to a pre-morbid level. She is now awaiting orthopaedic intervention to facilitate her mobility.

### Case study 2

A 42-year-old single man with moderate intellectual disability, autism and a history of affective psychosis in remission presented with urinary retention requiring catheterisation. This was attributed to anticholinergic side-effects of his psychotropic medications. These were therefore stopped. He later developed sepsis and needed to be treated with intravenous antibiotics. At this point, his estimated glomerular filtration rate had reduced dramatically (27 ml/min) and his urea and creatine increased (13.3 mmol/l and 240 umol/l respectively); as he also was on lithium, this had to be stopped to avoid toxicity. Following treatment, his kidney function tests came back to normal but his condition deteriorated and he displayed aggressive outbursts and insomnia. Given his recent history of urinary retention/renal failure, extreme aggression and a history of affective psychosis, he was subsequently treated with a new generation antipsychotic, aripiprazole, but this could not be continued because of allergic skin rashes. A few days following the cessation of aripiprazole he appeared vacant and confused, unable to communicate, and was rolling on the floor while kicking out in the air. Owing to extreme agitation, he needed benzodiazepine agents and intensive two-to-one support to ensure he did not sustain injury. The floor and walls of the room were covered with soft mattresses to prevent any skin breakdown due to friction on the floor. He had speech and language therapy input to prevent aspiration but soon stopped oral intake completely. With a working diagnosis of acute delirious state, he underwent extensive investigations including blood tests, lumbar puncture and brain scan, which all were reported as normal. His EEG at this stage reported generalised low-amplitude slow-wave activities in line with a mild diffuse cerebral dysfunction.

Since all investigations were within normal range, the clinical picture was attributed to a rapid withdrawal of his psychotropic medications and therefore a low dose of olanzapine (2.5 mg daily) was started. However, he deteriorated and his vital signs started to fluctuate. Blood investigations revealed extremely raised CK level (5369 iu/l). His white blood cell counts, kidney and liver function tests, however, were within normal range. Olanzapine was stopped and a working diagnosis of NMS was made. He received intravenous bromocriptine but even after CK level came back to normal, he did not show any improvement. During this time he presented as non-responsive, with decreased level of consciousness and some repetitive swinging movements of his arms and legs. All the investigations, including brain MRI scan, lumbar puncture and numerous blood/urine tests came back as normal. Swallowing difficulties with a high risk of aspiration resulted in the requirement of a radiologically inserted gastrostomy. He subsequently developed pneumonia and was placed on positive airway pressure for a short time. Treatment with intravenous antibiotics resulted in recovery of pneumonia but he developed severe diarrhoea owing to clostridium difficile.

At this stage it was felt that his condition could not be explained simply by a change in his medication or NMS and further investigations revealed positive anti-NMDA-receptor antibodies. Various investigations, including tests for other autoimmune encephalitides, tumour markers and chest, abdominal and pelvic CT scans were carried out to rule out an associated neoplasm, which has been reported to co-occur with anti-NMDA-receptor encephalitis in male patients,^12^ but the results came back negative. He was treated with methylprednisolone, after which he started to improve cognitively; however, he continued to go through latter stages of the disease and developed seizures and orofacial dyskinesia. He had another course of methylprednisolone and gradually, over a period of a few months, started eating and walking.

## Discussion

These two cases of anti-NMDA-receptor encephalitis, in individuals with intellectual disability and autism, presented with signs and symptoms of NMS and catatonia. In both patients the diagnosis was made with delay owing to the complexity of their presentation.

One of the characteristic features in Case study 1 was the presence of autonomic dysfunction which manifested with clammy skin, low-grade fever, persistent sinus tachycardia and fluctuation in blood pressure. These were unrelated to an underlying infection or dehydration. The patient also had short-lived episodes of central apnoea which confusingly presented itself similar to breath-holding attacks, which were first thought to be behavioural in nature. Autonomic instability in Case study 2 presented with episodes of urinary retention necessitating frequent catheterisations. Both patients developed frequent urinary and chest infections, muscle atrophy and contractures needing a multi-agency approach and intensive skin care to prevent pressure sores. During the first few months of their illness, both patients required two-to-one staffing support on a daily basis. In spite of intensive multi-agency support provided by the health and social care services, the experience was overwhelmingly traumatic and stressful for the families who had to cope with witnessing the patients going through a life-threatening and debilitating illness.

### Differential diagnoses

Anti-NMDA-receptor encephalitis can be mistaken for psychosis^13^ or catatonia.^14,15^ Concerns have been raised that catatonia is underdiagnosed. One study^16^ found that in Scotland the prevalence of catatonia varied depending on the diagnostic criteria used, ranging from 1.3 to 32%. In that study the prevalence of psychiatric patients demonstrating any catatonic signs was at least 7.9–19.1%. The most common catatonic signs were marked underactivity, echolalia, palilalia, marked overactivity and gegenhalten. In those with catatonic signs, the most common diagnoses were schizophrenia, schizoaffective disorder, dementia and non-psychiatric disorders (1.5%).

Malignant (lethal) catatonia presents with clouding of consciousness, autonomic instability, mutism, refusal to eat and drink, rigidity, waxy flexibility and posturing, and can be mistaken for NMS. Raised creatine kinase-skeletal muscles isoenzyme and leukocytosis are present in both conditions. It has therefore been suggested that, on the basis of the similarity of signs, symptoms and response to treatment, malignant catatonia and NMS should be considered to be the same disorder; NMS may also be understood as an antipsychotic-induced form of lethal catatonia.^17^ Interestingly, both NMS and catatonia might be seen in a patient at the same time, with one evolving into another in the course of illness.^18,19^

Catatonia resistant to benzodiazepine and electroconvulsive therapy has been treated with NMDA-antagonists (amantadine and memantine).^20^ Theoretically, however, these may exacerbate anti-NMDA-receptor encephalitis. Care should be therefore taken to avoid diagnostic overshadowing in people with autism and intellectual disability who have communication difficulties, as early treatment prevents mortality and long-term cognitive complications.^21^ A recent case report of catatonia in a deaf patient^22^ highlights this important issue in a vulnerable population with communication difficulties.

It is also important to be aware of other differential diagnoses such as viral encephalitis,^23^ a catatonic state induced by a psychiatric disorder or catatonia seen in people with autism spectrum disorder,^24^ substance misuse, serotonergic syndrome, heat stroke,^25^ other autoimmune encephalitides such as antiphospholipid syndrome,^26^ and catatonia induced by pernicious anaemia.^27^

This report highlights the complex presentation of anti-NMDA-receptor encephalitis in two patients with intellectual disability and autism. Whether or not people who have underlying brain pathology are more prone to develop this type of encephalitis or have a worse prognosis in comparison with the rest of the population needs further exploration through future case studies and multicentre research projects.
